# Precision Detection of Dense Plums in Orchards Using the Improved YOLOv4 Model

**DOI:** 10.3389/fpls.2022.839269

**Published:** 2022-03-11

**Authors:** Lele Wang, Yingjie Zhao, Shengbo Liu, Yuanhong Li, Shengde Chen, Yubin Lan

**Affiliations:** ^1^College of Electronic Engineering, College of Artificial Intelligence, South China Agricultural University, Guangzhou, China; ^2^Guangdong Laboratory for Lingnan Modern Agriculture, Guangzhou, China; ^3^National Center for International Collaboration Research on Precision Agricultural Aviation Pesticides Spraying Technology, Guangzhou, China; ^4^Department of Biological and Agricultural Engineering, Texas A&M University, College Station, TX, United States

**Keywords:** object detection, YOLOv4, MobileNetV3, data balance, plum

## Abstract

The precision detection of dense small targets in orchards is critical for the visual perception of agricultural picking robots. At present, the visual detection algorithms for plums still have a poor recognition effect due to the characteristics of small plum shapes and dense growth. Thus, this paper proposed a lightweight model based on the improved You Only Look Once version 4 (YOLOv4) to detect dense plums in orchards. First, we employed a data augmentation method based on category balance to alleviate the imbalance in the number of plums of different maturity levels and insufficient data quantity. Second, we abandoned Center and Scale Prediction Darknet53 (CSPDarknet53) and chose a lighter MobilenetV3 on selecting backbone feature extraction networks. In the feature fusion stage, we used depthwise separable convolution (DSC) instead of standard convolution to achieve the purpose of reducing model parameters. To solve the insufficient feature extraction problem of dense targets, this model achieved fine-grained detection by introducing a 152 × 152 feature layer. The Focal loss and complete intersection over union (CIOU) loss were joined to balance the contribution of hard-to-classify and easy-to-classify samples to the total loss. Then, the improved model was trained through transfer learning at different stages. Finally, several groups of detection experiments were designed to evaluate the performance of the improved model. The results showed that the improved YOLOv4 model had the best mean average precision (mAP) performance than YOLOv4, YOLOv4-tiny, and MobileNet-Single Shot Multibox Detector (MobileNet-SSD). Compared with some results from the YOLOv4 model, the model size of the improved model is compressed by 77.85%, the parameters are only 17.92% of the original model parameters, and the detection speed is accelerated by 112%. In addition, the influence of the automatic data balance algorithm on the accuracy of the model and the detection effect of the improved model under different illumination angles, different intensity levels, and different types of occlusions were discussed in this paper. It is indicated that the improved detection model has strong robustness and high accuracy under the real natural environment, which can provide data reference for the subsequent orchard yield estimation and engineering applications of robot picking work.

## Introduction

Plum is a characteristic fruit in South China. Its fruit is small, densely distributed, and easily blocked by other plums or branches and leaves. Plum maturity identification and picking tasks are completed manually in the current plum orchards. At present, labor costs have unprecedentedly increased, and the proportion of labor costs in total costs is also increasing, with the increase reaching up to 12–15% in 2019 (Fu et al., 2020a). In precision agriculture, labor shortage and aging labor have posed barriers to the development of the fruit industry. Considering the above, mechanized and intelligent intensive plum picking is an indispensable part of the development of the whole fruit industry.

In recent years, relevant scholars have carried out a series of research on recognizing and detecting fruits, such as apples and citrus in precision orchards (Liao et al., 2017; Wajid et al., 2018; Gurubelli et al., 2019; Mo et al., 2021). Lin G. et al. (2020) adopted partial shape matching and probabilistic Hough transform to detect fruits in the natural environment. Fu et al. (2019) achieved the fine detection of bananas by combining color, texture features, and Support Vector Machine classifier. He et al. (2020) put forward a green citrus detection method based on the deep boundary box regression forest by fusing multiscale features of color, shape, and texture. Zhao et al. (2016) combined AdaBoost classifier and color analysis to detect tomatoes in the greenhouse scene. In summary, these studies discussed previously mainly combined the traditional image processing methods and the basic characteristics of fruit color and texture. However, the data processing required a comprehensive analysis of multiple features, complex processing procedures, and poor real-time detection, which were difficult to meet the requirements of orchard information management and robotic picking.

With the rapid development of machine learning, the deep convolutional neural network (CNN) has shown excellent performance in fruits detection. Its high extraction of high-dimensional targets features makes it possible to recognize in complex environments. There are two-stage detection methods, such as Fast RCNN (Girshick, 2015) and Faster R-CNN (Ren et al., 2016). These target detection models based on the region suggestion method adopt the final layer of the CNN to predict. Xiong et al. (2018) employed the Faster R-CNN method to detect green citrus under different illumination and sizes, and the accuracy rate reached 77.45%. Zhang et al. (2020) developed three apple recognition algorithms based on Faster R-CNN, with mean average precision (mAP) of up to 82.4%. Fu et al. (2020b) established an algorithm that is composed of ZFNet and VGG16 of Faster R-CNN architecture to detect apples in dense-leaf fruit wall trees, and the results showed that the removal of background trees with a depth filter improved fruit detection accuracy by 2.5%.

In addition, single-stage target detection methods, such as SSD (Liu et al., 2016) and YOLO (Redmon et al., 2016; Redmon and Farhadi, 2017, 2018), have been widely used because of their high accuracy and detection efficiency. Xue et al. (2018) adopted the YOLOv2 network to identify immature mango, which improved the detection rate while maintaining accuracy and generalization capability. Some researchers (Liu and Wang, 2020; Wang and Liu, 2021a,b) proposed the improved network models of YOLOv3 to detect the diseases and pests of greenhouse tomatoes. The proposed detection algorithm had strong robustness and high accuracy in complex orchard scenes. Tian et al. (2019) designed an improved YOLOv3 model to detect apple at different growth stages in the orchard. Kuznetsova et al. (2020) proposed a YOLOv3 apple detection algorithm with special pre-processing and post-processing. Li et al. (2020) employed the MobileNet-YOLOv3 model to detect dragon fruit in the orchard. Wu et al. (2021) proposed an improved YOLOv3 model based on clustering optimization. Liu et al. (2021) proposed a YOLOv3-SE improved method for winter jujube fruit recognition under natural environment. The mAP of the improved model increased by 2.38∼4.81% through the analysis of detection effects under different lighting conditions, occlusion, and maturity. Ji et al. (2021) proposed an apple detection method based on the improved YOLOv4, which could accurately locate and detect apples in various complex environments. Although the YOLO series networks have shown excellent performance in fruit recognition, it is difficult to detect small targets in deep feature maps due to the loss of spatial and detailed feature information. Due to the large number of model parameters, it is a very challenging task to deploy on the devices with limited resources and achieve the goal of real-time reasoning.

Compared with apple, citrus, mango, and other fruits, plum trees are mostly planted on hillsides, and their fruit growth environment is full of complexity and uncertainty. In modern precision orchards, it is more difficult to detect small targets owing to the presence of complex noise disturbance, such as changing illumination and branch and leaf occlusion. In addition, the cluster growth of the plum itself and the mixing of different maturity lead to the poor performance of existing algorithms in plum detection (Gao X. et al., 2021). Jang et al. (2021) tried to use 3D images and MATLAB R2018a to detect plums and size estimation, and this method achieved an average recognition rate of 61.9%. Pourdarbani et al. (2019) established different classifiers and majority voting rules to compare the effects of 12 different light intensities on plum images segmentation in the natural environment, and the experimental results showed that the correct classification results of the majority voting method excluding LDA were better than those of the composition method. Brown and Sukkarieh (2021) presented two datasets gathered during a robotic harvesting trial on 2D trellis plums and used them to benchmark on the four deep learning object detection architectures. Although many researchers have done extensive work on the detection of plums, the accuracy and robustness in different scenes still need to be further improved. So far, no study has been conducted on deep learning methods to detect dense plums in natural environments. The resources that fruit-picking robots can use in the orchard are limited. Therefore, it is necessary to explore an efficient and accurate plum recognition algorithm according to actual needs.

Aiming at the growth characteristics of plum fruit, this paper took advantage of the YOLOv4 network in target detection and combined it with the MobileNetV3 lightweight network. In the feature fusion structure, deep separable convolution was introduced to replace standard convolution, and a new convolution layer was introduced to increase the recognition performance of the model for dense small targets. Meanwhile, the Focal loss function was added to balance the contribution of different samples to the total loss. The proposed method is compared and evaluated with the other three target detection networks in different scenes to provide a reference for the yield estimation of plum and the rapid recognition of picking robots.

## Materials and Methods

### Materials

#### Image and Data Acquisition

**FIGURE 1:**
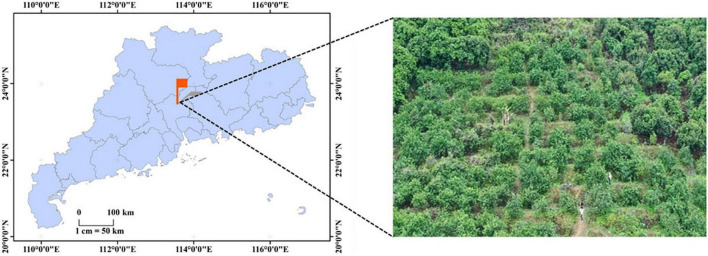
Location of images acquisition site.

The experimental collection site is located in a plum orchard (23.55N, 113.59E) in Conghua District, Guangzhou City, Guangdong Province, China. The geographical location of the image acquisition is shown in Figure 1. The sampling device in this study is a high-resolution smartphone with a camera parameter of 40 million pixels, the exposure parameter is automatic, and the objective focus system is set to autofocus mode.

The sampling objects were plums. To collect as much information about plums in the natural environment as possible, the experimenter simulated the image capture module of the picking robot, and the handheld collection device continuously changed the shooting angle and shooting distance, hoping to collect RGB images of different colors, postures, sizes, backgrounds, and density. The experimental samples were obtained in two batches. The photographs were taken on April 24, 2021, which was a sunny day. The weather changed from light rain to cloudy from May 3 to 4, 2021. The plums were in the middle of maturity during these sessions. Most mature plums’ color is red, and some immature plums’ color is cyan. In total, 1,890 original images were collected under different scenes. Mature and immature plums were included in the photographs. The overall quality of the image could meet the requirements of target detection by making a visual quality assessment on the collected image data.

#### Dataset Production

The collected plum images have 3,968 × 2,976 pixels. However, the high pixel will prolong the training and processing time. This study adopted a bicubic scaling algorithm to scale image pixels to 1,920 × 1,440.

**FIGURE 2:**
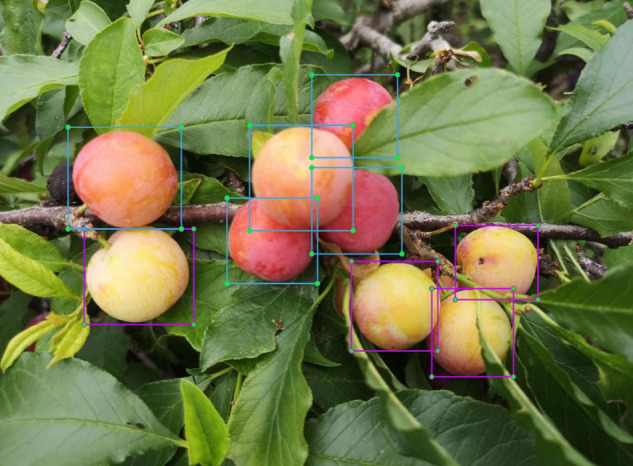
Data annotation example: the blue box represents mature plums, and the purple box represents immature plums.

The Label Img, an image annotation tool, was used for manual annotation to obtain the ground truth for subsequent training. As shown in Figure 2, the wholly exposed plums are marked by cutting the outer part to the inside of the rectangular frame. For occluded or conglutinated plums, only the exposed parts of the image are marked. The unmarked processing was performed when the part of the image boundary or the degree of occluded plums was less than 10%. The annotation information was saved in the format of the PASCAL VOC dataset. The maturity was manually judged and marked as two types of plums, mature (plum) and immature (raw_plum).

For the marked 1,890 plum images, the original dataset was divided into the training set, validation set, and test set, where the ratio of training set to test set is 8:2. The validation set is randomly selected from 10% of the training set and does not participate in training. The training set was divided into three sub-datasets according to different collection times. Among them, sub-dataset 1 was composed of 368 image data collected on April 24, 2021, sub-dataset 2 was composed of 400 images collected on May 2, 2021, and sub-dataset 3 was composed of 744 image data collected on May 3, 2021. Table 1 shows the data before data balance.

**TABLE 1:** The number of datasets before and after augmentation.

Collection date	Dataset	Processing method	Number of pictures	Mature labels	Immature labels
April 24, 2021	Sub-dataset 1	Before augmentation	368	1,353	3,287
		After augmentation	4,416	16,236	39,444
May 2, 2021	Sub-dataset 2	Before augmentation	400	2,347	258
		After augmentation	2,400	9,388	1,548
May 3, 2021	Sub-dataset 3	Before augmentation	744	4,634	317
		After augmentation	4,464	27,804	1,902
	Total	Before augmentation	1,512	8,334	3,862
		After augmentation	11,280	53,428	42,894

According to the number of plums in 1,890 labeled images, there are 10,441 mature and 4,754 immature plums labels. The proportion between the two is close to 2.2:1. It can be found that there is a larger data imbalance between the number of mature plums and immature plums. If the network model is trained directly, it will have poor recognition performance for immature plums, resulting in the degradation of model detection ability. Therefore, it is necessary to take some measures to balance the dataset to improve the recognition ability of the model for immature plums.

#### Data Augmentation Method Based on Category Balance

##### Automatic Data Balancing Method Based on Category

Aiming at the imbalance mentioned above, this paper proposed an automatic data balancing method based on category to optimize the dataset so that the number of categories before the network model training is the same as possible (Gao J. et al., 2021). This method needs to obtain the quantitative values of all categories first, compare and select the category with the largest amount of data, and then sequentially expand the quantitative values of other categories to approach the largest category. The specific steps are as follows:

i. Suppose there is a dataset *S* = [*M*_1_, *M*_2_,, *M*_*i*_][*N*_1_, *N*_2_, …, *N*_*j*_]^*T*^, where *M_i_* denotes the number of types of samples in the dataset, and *N*_*j*_ denotes the number of samples in each category;
ii. Compare the sample quantity values of all categories in the dataset *M*_*i*_*N*_*j*_ and find the maximum value *M*_*i*_*N*_*jmax*_;
iii. Use *M*_*i*_*N*_*jmax*_ to divide by the sample quantity value *M*_*i*_*N*_*j*_ of the remaining category in turn, and then division C is obtained. The calculation is given in Equation 1:

(1) $$C=\frac{M_{i}N_{jmax}}{M_{i}N_{j}}=\left[\left\{c_{1},c_{2},\ldots ,c_{i-1}\right\}\right]$$

iv. Choose a data quantity expansion method, and the data quantity of residual categories become large according to division C so that the number of samples of all categories is expanded to the maximum value, and MiNj′ is obtained, and finally, the quantity proportion of each category is close to 1;
v. The final output is the expanded dataset T=[M1,M2,,Mi][N,1′N,2′,N]jmax′T.

According to the automatic data balancing algorithm, the number of mature groups is divided by the number of immature groups in the whole dataset, and the remainder is rounded down to get 2. Since there are different proportions of mature plum and immature plum labels in each sub-dataset, it is necessary to balance the whole sub-dataset in data balancing. Therefore, only one data amplification of sub-dataset 1 can ensure that the overall proportion of immature and mature plums in the dataset is close to 1.

##### Data Augmentation

To prevent overfitting or non-convergence phenomenon caused by too little training data, this study randomly combines common data augmentation methods and performs data augmentation processing on the train set, such as Gaussian blur, random rotation, random cutting off part of the image, histogram equalization, random brightness adjustment, salt, and pepper noise (Huang et al., 2020; Wu et al., 2020). The dataset is enhanced five times through the multiple random combinations of the above methods. The enhanced dataset is shown in Table 1. At the same time, thanks to the data balance method adopted, the proportion of mature and immature plums in the training set has changed from 2.2:1 to 1.2:1 so that the number of different categories of the dataset is similar.

### Methodologies

#### YOLOv4 Model

The YOLO series target detection models are widely used in industry and scientific research due to their excellent speed and detection accuracy performance. Bochkovskiy et al. (2020) proposed the YOLOv4 model based on YOLOv3, which has better recognition performance and faster speed. It can carry out end-to-end object prediction and classification. It is one of the most high-performance target detection methods at present. Compared with the YOLOv3 network, the main improvements of YOLOv4 include: (1) The Mosaic data augmentation method is designed, and the input images are merged by random clipping, scaling, and spatial arrangement. At the same time, training techniques, such as the learning rate cosine annealing attenuation method are used. (2) The new backbone network and activation function are used to enhance the feature learning ability of the network. Meanwhile, DropBlock regularization is used to alleviate the overfitting problem. (3) The Spatial Pyramid Pooling (SPP) module and Path Aggregation Network (PANet) structure are introduced. The PANet structure is used to transfer semantic features from top to bottom, and the feature pyramid is designed to transfer location features from bottom to top and aggregated through the backbone layer to improve the ability of network feature extraction. (4) The CIOU loss function is introduced to increase the width-to-height ratio information of the bounding box and enhance the robustness. The DIOU_nms prediction box screening mechanism is used to improve the screening performance of overlapping targets.

A YOLOv4 network model mainly consists of the backbone, neck, and head networks. The backbone network is the CSPDarknet53 network, composed of 5 modules from Center and Scale Prediction 1 (CSP1) to CSP5, and each module is alternately stacked with CSPX and synthesis module of convolution, batch regularization, and Mish activation function (CBM) modules. After the input picture passes through the backbone network, the feature maps with three scales of 52 × 52 × 256, 26 × 26 × 512, and 13 × 13 × 1,024 are obtained. The feature maps of different scales contain semantic information of different dimensions. For the 13 × 13 × 1,024 feature layer, the maximum pooling of different scales is performed in the SPP structure to increase the receptive field of the network. After that, the three feature layers obtained are input into the PANet for a series of feature fusion, and finally, three detection heads of 13 × 13, 26 × 26, and 52 × 52 are output, respectively. Through decoding and non-maximum suppression of the detection head, the final prediction box is generated to detect the objects of different scales.

#### Depthwise Separable Convolution

Depthwise separable convolution is a lightweight convolution method, which can effectively reduce the amount of calculation compared with standard convolution. For the feature map with an input size of (*D*_*x*_, *D*_*y*_, *M*), the principle of depthwise separable convolution is to first separate Channel-by-channel convolve M convolution kernels of size (*D*_*k*_, *D*_*k*_) and each channel of the input feature map, and then, obtain a feature map where the input channel is equal to the output channel. Finally, N convolution kernels with size (1, 1) are used to pointwise convolution the feature map, and a new feature map (*D*_*w*_, *D*_*h*_, *N*) is obtained. Under the premise that the convolution characteristics are similar to the standard convolution performance, depthwise separable convolution can effectively reduce the network model’s parameter amount and calculation amount. Furthermore, the speed of model training and reasoning is significantly accelerated.

#### Backbone Network

To pursue the model’s high accuracy and better performance, many scholars have deepen the number of layers of the network model. However, this scheme has some drawbacks, such as increasing the number of parameters of the model, aggravating the calculation of the model, and reducing the operation efficiency of the model, which make it difficult to deploy on devices with limited computing resources. In picking robot operation, real-time performance is one of the most critical performance indicators, so it is necessary to lightweight the network reduce the calculation amount of the model. Although the CSPDarknet53 network used in the YOLOv4 model has strong feature extraction performance, the model is complicated and requires more computation.

**FIGURE 3:**
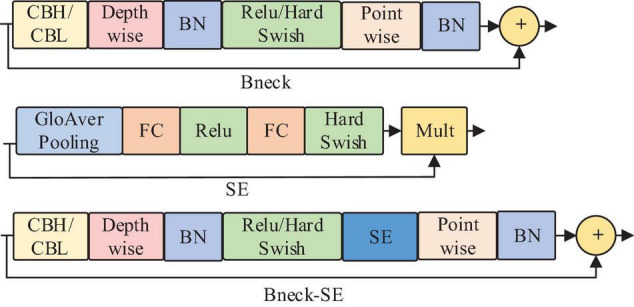
Structure diagram of Bneck.

The MobileNetV3 network (Howard et al., 2019) combines deep separable convolution, MobileNetV2’s inverted residual structure with linear bottleneck (Howard et al., 2018), and MnasNet’s lightweight attention model based on the squeeze and excitation structure (Hu et al., 2018). MobileNetV3 constructs the network by combining these layers as construction Bneck, which successively passes through 1 × 1 ascending convolution, 3 × 3 depthwise separable convolution, and 1 × 1 dimension reduction convolution. The structure is shown in Figure 3. Moreover, the lightweight attention mechanism of the SE structure is introduced further to improve the feature extraction ability of the model. Eventually, the whole network structure is composed of Bneck stacks. Wherein CBL and CBH represent the synthesis modules of convolution, batch regularization, and LekeyReLU or h-swish activation functions; BN represents Batch Normalization; FC represents Full Connection; SE represents squeeze-and-excitation.

### The Proposed Algorithm

**FIGURE 4:**
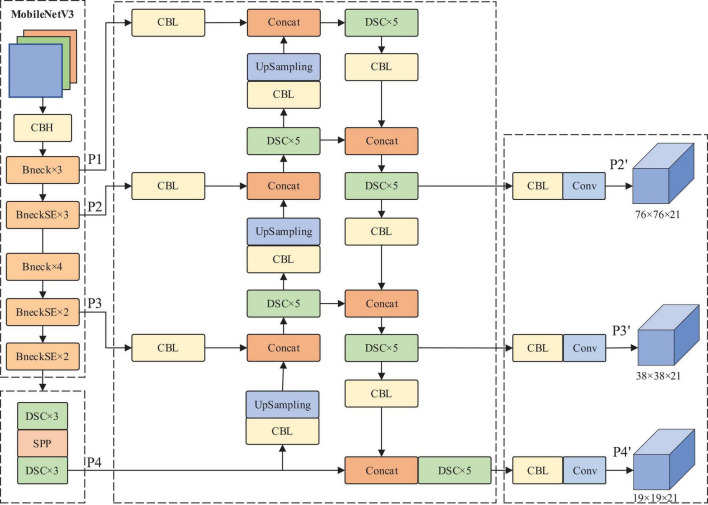
The structure diagram of improved YOLOv4.

To effectively identify dense plums, this paper chose 608 × 608 as the input size of the YOLOv4 model, and MobileNetV3 was used to replace the CSPDarknet53 backbone network of the original model, which could effectively reduce the number of parameters of the model backbone network. The depthwise separable convolution was employed to replace the standard convolution in the original PANet to further reduce the number of model parameters. The model convolution module can obtain higher feature information through multiple down-sampling. However, when the feature layer with higher semantic information in the feature fusion network is up-sampled and fused, the convolution module will lose a certain amount of information, so the detection accuracy of small targets will be reduced. Therefore, this paper introduced the 152 × 152 × 24 layer to obtain more abundant shallow information to achieve fine-grained detection of small target objects. Due to the small pixels of plums in the whole image, the model will pay too much attention to the simple training samples and ignore the samples that are difficult to classify. Therefore, this paper introduced the Focal loss function to measure the contribution of difficult classification and easy classification to the total loss. The combined loss function of Focal loss and CIOU loss was designed as the loss function of the improved model. On this basis, this paper used transfer learning to train the model. Through the two-stage learning, the model’s generalization performance can be further improved, and the dense plums can be identified quickly and accurately. The improved YOLOv4 model structure is shown in Figure 4. Among them, Conv means convolution, and DSC means depthwise separable convolution. DSC × 5 indicates that five depthwise separable convolution operations are required.

#### Multiscale Fusion Network Structure

In this study, the YOLOv4 algorithm was improved to solve the problem of insufficient feature extraction in dense plums recognition. When the input image size selected by the YOLOv4 model is 608 × 608, the feature layer responsible for predicting dense small targets is 76 × 76, and each feature grid’s corresponding receptive field size is 8 × 8. When the input picture resolution is 1,920 × 1,080, the corresponding long edge is 25 through YOLO grid compression. That is to say; when the target feature size is less than 25 × 25 pixels, the target feature information cannot be effectively learned.

To extract the feature information of dense plums as much as possible, this study improved the network model of YOLOv4. Four feature layers were output from the backbone network MobileNetV3, namely P1 (152 × 152), P2 (76 × 76), P3 (38 × 38), and P4 (19 × 19). Among them, the P4 feature layer has the largest receptive field, which is suitable for large-scale target detection, and the receptive field of the P3 feature layer is suitable for medium-scale target detection. P2 is up-sampled and fused with the P1 feature layer, a relatively rich shallow layer can be obtained, which enables to achieve the fine-grained detection of small target objects. In the process of feature propagation, P4 is still obtained through the SPP structure. This study combines the feature layers P4, P3, P2, and P1 with different pyramid-level feature maps through up-sampling in the feature pyramid network (FPN) structure. Each feature layer is transformed by convolution and up-sampling to obtain the same scale and channel number as the previous feature layer and then stacked and fused with the previous feature layer to obtain a feature map with more abundant information. The improved network structure is shown in Figure 4.

The four feature layers from the FPN feature fusion output were pruned to prevent the network from being too redundant. The specific operation was that the 152 × 152 scale feature layer output by FPN is no longer the predicted output and directly up-sampled in the PANet structure. Therefore, the improved algorithm maintains the prediction YOLO head of three scales, namely P2’ (76 × 76), P3’ (38 × 38), and P4’ (19 × 19).

Furthermore, the depthwise separable convolution was introduced into the PANet structure to replace the partial convolution of the original network. The improvement can effectively compress the number of network parameters and the amount of calculation.

#### Improvement of the Loss Function

The loss function of YOLOv4 consists of CIOU bounding box loss, classification loss, and confidence loss. The calculation method is shown in Formula (2)–(6):


(2) $$L=L_{CIOU}+L_{class}+L_{conf}$$

(3) $$L_{CIOU}=1-IOU\left(A,B\right)+\frac{\rho^{2}\left(A_{ctr},B_{ctr}\right)}{c^{2}}+\alpha \nu$$

(4) $$\nu =\frac{4}{\pi^{2}}{\left(\tan^{-1}\frac{w{}^{gt}}{h{}^{gt}}-\tan^{-1}\frac{w}{h}\right)}^{2}$$

(5) $$\alpha =\frac{\nu }{\left(1-IOU\right)+\nu }$$

(6) $$IOU=\frac{\left|A⋂B\right|}{\left|A⋃B\right|}$$

Among them, A and B represent the area of the prediction frame and the actual frame, and the range of IOU is [0,1]; w^gt^ represents the width and height of the actual frame; w and h represent the width and height of the prediction frame; A_ctr_ and B_ctr_ represent the coordinates of the predicted box’s center points and the actual box; ρ represents the Euclidean distance; c is the diagonal length of the smallest bounding box C composed of A and B; ν represents the penalty term.

Owing to the small physical size of plums and fewer pixels occupied in the image, when there are single, occluded, and densely stacked plums in an image, the model will automatically pay attention to and train single or easy-to-recognize simple samples, ignoring adhesion, and other difficult to classify samples. Therefore, it is necessary to find an appropriate loss function to balance the contribution of hard-to-classify and easy-to-classify samples to the total loss.

The Focal loss focused on hard-to-classify samples during the training process without affecting the original detection speed. Formula (7) of this function is as follows (Li et al., 2020; Long et al., 2021; Zhao et al., 2021):


(7) FL(pt)={-αt(1-pt)γln(pt),ify=1-(1-αt)ptγln(1-pt)},otherwise

Where *y* is the number of sample labels; *p*_*t*_ represents the probability of belonging to the plum category; α_*t*_ is the coefficient of balancing the weight of positive and negative samples, 0 < α_*t*_ < 1; γ is the modulation parameter for complex samples.

This paper employed Focal Loss to replace class loss in the original loss function. Taking the prediction of simple mature plum as an example, when the *p_t_* value is small, and the (1−*p*_*t*_)^γ^ value is close to 1, and its loss is almost unaffected. When *p_t_* is large and close to 1, it indicates that the classification prediction result is better. If it is not corrected, it will easily interfere with the optimization direction of the model. After introducing Focal Loss, when *p_t_* is larger, (1−*p*_*t*_)^γ^ is smaller. With the increase of γ, the faster the rate of simple sample reduction is adjusted, and the lower the proportion of simple samples in the total loss value. Therefore, the network model can focus more on hard-to-classify samples by introducing Focal Loss.

#### Plum Model Training Based on Transfer Learning

The hardware and software platform for model training was configured as follows: CPU is AMD R5-5600X 3.7 GHz, memory is 32 GB, storage SSD is 512 GB, display card is NVIDIA RTX2060S, display memory is 8 GB, the operating system is Windows10, CUDA version is 10.1, Python version is 3.7, and the PyTorch version is 1.6.

In this experiment, the input image pixels are 1,920 × 1,440. The K-means algorithm was used to generate the anchors’ coordinate frame iteratively, and the Adam optimizer was used. The improved loss function was used to train the model. In addition to offline augmentation methods, Mosaic data augmentation was used in the training process to enrich the background of the detected objects further, strengthen the cognition of the network model on plum characteristics, and enhance the robustness and generalization performance of the model. The initial value of the learning rate was set to 10^−4^, and the cosine annealing learning rate was optimized and updated during the training process.

**FIGURE 5:**
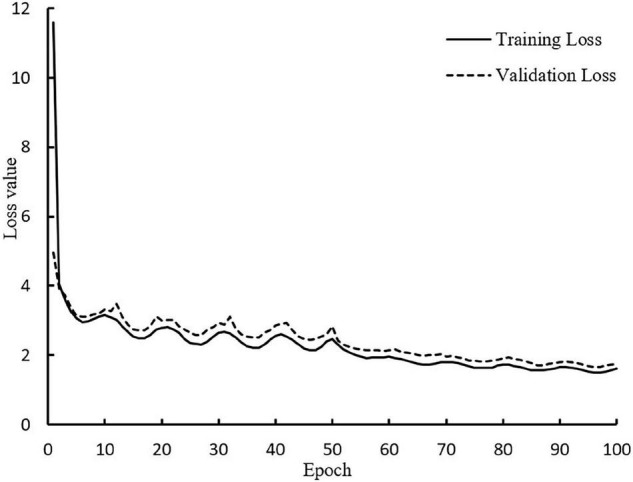
Loss curve during training process.

To speed up the convergence of the model, this paper adopted the transfer learning method for training. The training was divided into two stages, and the whole stage was trained for 100 epochs. For the first half of the stage, the pre-training weight of the MobileNetV3 network was loaded, and the backbone feature extraction network of the model was trained 50 epochs by freezing. The initial value of the learning rate was set to 1 × 10^−3^, and the batch size was set to 16. This operation can accelerate the convergence speed and prevent the pre-training weight from being destroyed. For the second half of the stage, the backbone feature extraction network was unfrozen, and the entire model was further trained for 50 epochs with an initial learning rate of 1 × 10^−4^, and the batch size was set to 8. The convergence of the entire model was accelerated through two stages, and the training time of the model was shortened. In the training process, validation is performed after each epoch of training, and there is no overlap of the validation and test set. The weight file of each round of training was saved, and the loss values of the training set and validation set were saved. The loss value curves of the training set and validation set of the improved model in this paper are shown in Figure 5.

## Experimental Results and Comparative Analysis

### Model Evaluation Indicators

To objectively measure the target detection effect of the model on dense plums, the precision (P), recall (R), harmonic average F1 value (F1), average precision (AP), mAP, the number of network parameters, the size of the weight, and the detection speed were used to evaluate the trained model. The Intersection over Union (IoU) value was 0.5 in the experiment. The calculation formulas of P, R, F1, AP, and mAP are shown in formulas (8–12).


(8) $$P=\frac{TP}{TP+FP}$$

(9) $$R=\frac{TP}{TP+FN}$$

(10) $$F1=\frac{2PR}{P+R}$$

(11) $$AP=\int_{0}^{1}P\left(R\right)dR$$

(12) $$mAP=\frac{\int_{q=1}^{Q}AP\left(q\right)}{Q}$$

Among them, *TP* represents the number of correctly detected plums; *FP* represents the number of misclassified plums; *FN* represents the number of missed plums; *F*1 represents the harmonic average of accuracy and recall. When *F*1 is closer to 1, the model is better optimized. *AP* represents the area composed of the *PR* curve and the coordinate axis. The higher the *AP* value is, the better the performance of the target detection algorithm is. The *mAP* represents the AP average of multiple categories, and its value represents the general detection performance of the algorithm for different categories.

Detection speed refers to the length of the model detection time, which was used to evaluate the real-time performance of the detection models. It is usually measured by the number of frames per second (FPS). The larger the FPS, the faster the model detection speed. FPS refers to the number of images processed per second in this paper.

### Data Balance Comparison Experiments

This study selected the improved model based on YOLOv4 to train the plum data before and after the data balance. The same test set was selected to detect, and the evaluation index results are shown in Table 2. The data balance had little effect on the recognition rate of mature plums, which were both remained above 90%. Compared with the recognition rate of plums before data balance, the recognition rate of immature plums after balance increased by 6.11%, and the mAP of the test set also increased from 86 to 88.72%, with an increase of 2.72 percentage points. Overall, the recognition gap of plums with different maturity levels is alleviated, and the robustness of the model is enhanced.

**TABLE 2:** Comparison of recognition effect of the improved model before and after data balance.

Dataset types	Types Name	Plum AP	Raw_plum AP	mAP
Unbalanced data	A dataset	91.77%	80.23%	86.00%
Balanced data	B dataset	91.10%	86.34%	88.72%

**FIGURE 6:**
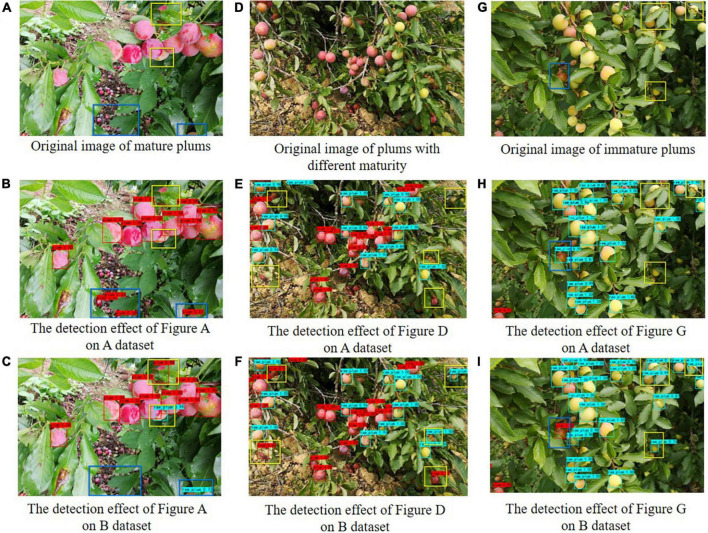
The comparison of detection effect of plum images before and after improved data balance.

Figure 6 shows the comparison of detection results before and after data balancing in different scenes, where A dataset represents the plum detection effect before data augmentation and B dataset represents the plum detection effect after data augmentation. By comparing the detection results before and after the data augmentation, we used the yellow frames to find out the missing plums in the (B, E, and H) image and marked the specific area in the original and the two types of detection images. Similarly, we used the blue frames to mark the specific areas where the plum was mistakenly detected.

A comprehensive comparison shows that the model after data balance has significantly improved the detection accuracy of immature plums, which indicates that the model’s ability to identify small sample features has been strengthened by improving the ratio of mature and immature plums. Meanwhile, the data-balanced model has improved the misdetection detection and missed detection of plums in scenes occluded by leaves and branches. In conclusion, the experimental results show the effectiveness of the data balance method.

### Comparative Experiments of Different Detection Methods

To evaluate the detection superiority of the improved model, the dataset made in this paper was trained by different target detection algorithms. After the training was completed, the test work was performed on the same testing sample sets. The AP, mAP value, model size, and detection speed of the four methods are shown in Table 3. Overall, the four models all had higher mAP for plums. Significantly, the improved YOLOv4 model was 1.59, 3.07, and 3.46 percentage points higher than the original YOLOv4, YOLOv4-tiny, and MobileNet-SSD, respectively, in the detection results of mature plums. Compared with the other three models, the improved YOLOv4 model increased by 2.59, 4.83, and 7.31 percentage points in the detection results of immature plums. Compared with the original YOLOv4 model, the improved YOLOv4 network model has a relatively simple structure, the model size of the improved YOLOv4 is compressed by 77.85%, which is only slightly more than two times the combined model size of the YOLOv4-tiny and MobileNet-SSD. Moreover, the parameters is only 17.92% of the original YOLOv4’s. The improved YOLOv4 network model is 112% faster than the original one in the terms of detection speed. In summary, the improved method presented in this paper shows the optimal detection performance for dense plums among the compared methods.

**TABLE 3:** Comparison of detection results of different architectures.

Architecture	Plum AP	Raw_plum AP	mAP	Model size	Parameters	FPS
YOLOv4	88.99%	83.95%	86.47%	244 MB	61.38 M	20.03
YOLOv4-tiny	87.51%	81.71%	84.61%	22.4 MB	5.77 M	112
MobileNet-SSD	87.12%	79.23%	83.18%	24.7 MB	5.98 M	82.84
Improved YOLOv4	90.58%	86.54%	88.56%	54.05 MB	11.00 M	42.55

### Comparative Experiment Under Different Light Conditions

**FIGURE 7:**
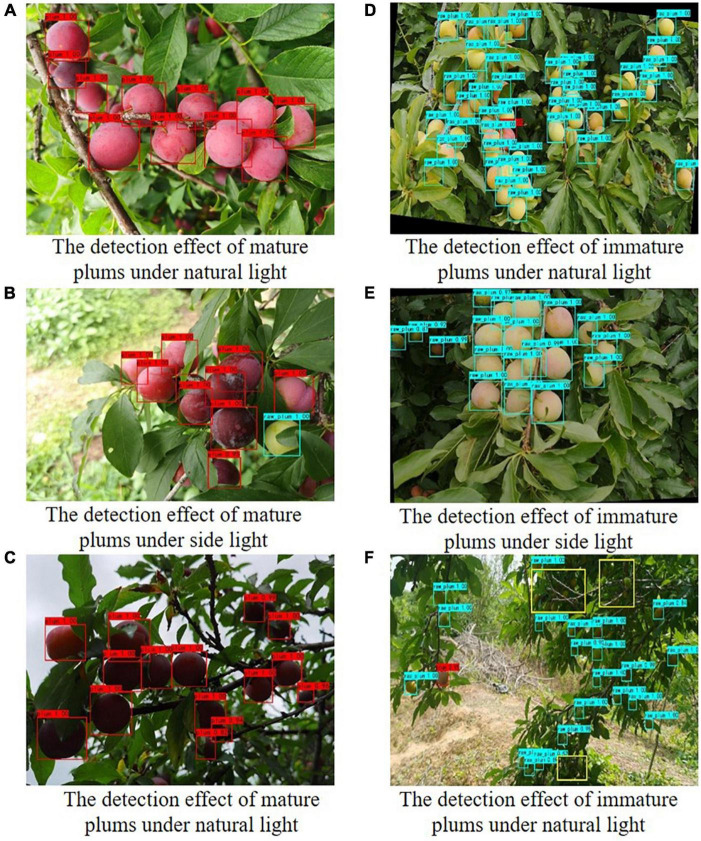
Plum detection effect pictures under different light conditions.

The visual system of the fruit picking robot is susceptible to the influence of different lighting conditions in the natural environment when it collects videos or images, which affects the change of recognition accuracy. Under natural lighting conditions, the image is bright and dark, and plum contours are clear. Under backlight conditions, the overall image is dark, and plum contours are not evident. Under sidelight conditions, plums have uneven brightness. Therefore, 40 additional plum images were randomly selected under natural light, side light, and backlight to form a new test set C. The evaluation performance index results are shown in Table 4, and the detection results are shown in Figure 7.

**TABLE 4:** Evaluation results of plum test set under different light conditions.

Light conditions	Classes	P	R	F1	mAP
Natural light	plum	90.32%	88.19%	0.89	94.53%
	raw_plum	89.41%	91.69%	0.91	
	mean value	89.87%	89.94%	0.9	
Side light	plum	88.29%	89.09%	0.89	94.86%
	raw_plum	93.07%	92.61%	0.93	
	mean value	90.68%	90.85%	0.91	
Back light	plum	90.14%	80.33%	0.85	86.75%
	raw_plum	92.36%	81.46%	0.87	
	mean value	91.25%	80.90%	0.86	

It can be seen from Table 4 that the improved model maintains a high accuracy rate for plum detection results under different light, but the detection results are discrepant under different angle light conditions. Among them, the model has a slight decrease in performance under backlight conditions. Compared with natural light and side light conditions, the mAP value of the backlight is lower by 7.78 and 8.11%, respectively. Thanks to the clear texture of the plum under the conditions of natural and sidelight, the improved model can obtain higher recognition accuracy. However, the backlight condition has a certain interference effect on image feature extraction.

Figure 7 shows the comparison of the detection effects of plum images under different lighting conditions. It can be seen from Figures 7A,D,B,E that the plum has clear texture and uniform surface light intensity under natural light and sidelight. The difficulty of image detection is relatively small. Even the plum target at a distance can be detected. In the backlight, the image clarity is insufficient, and the color of mature plum fruit is dark red. Moreover, the color discrimination between immature plum and background (such as, branches or leaves) decreases, so a small amount of missing detection occurs. Overall, the improved model still maintains a high recognition accuracy in natural orchards.

### Results and Analysis Under Dense Occlusion in Orchards

#### A Comparison Experiment of Plum Images With Different Density

We randomly selected some images with different densities for comparative experimental detection. If an image contains 10–20 plums, it is considered a moderately dense image. If there are more than 20 plums in the image, it is highly dense. Four architectures methods were used to test and compare the experimental results and detection results.

**FIGURE 8:**
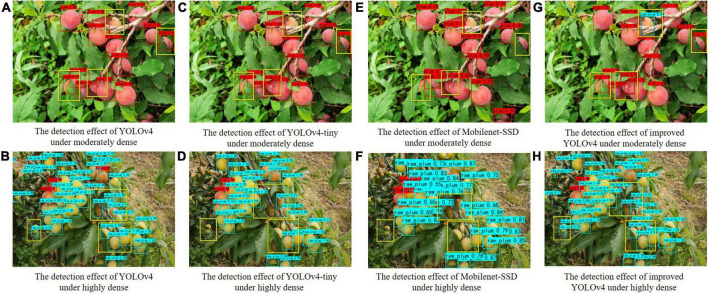
Plum detection effect pictures under different dense conditions.

As can be known from Table 5, the accuracy of moderately dense plum images is higher than that of highly dense plum images, mainly due to the severe occlusion of highly dense plums, unclear fruit edges, and lack of texture features. By comparing the mAP of the four target detection models, the improved YOLOv4 has the highest mAP, the moderately dense recognition mAP reaches 89.30%, and the highly dense recognition mAP reaches 84.75%. The mAP gap between the two densities when compared showed that MobileNet-SSD has the largest mAP gap, exceeding 10%. The mAP gap of the improved model in the paper is the smallest, with a gap of only 4.55%. This shows that the improved model has a better detection effect for plums with different densities, and the improved model can narrow the detection gap of plums with different densities. Compared with other models, the improved model has a lower missed recognition rate and can recognize more plums, as shown in Figure 8. The experimental results show that the improved method in this paper has better detection accuracy, which indicates that the improved multiscale fusion structure can extract more valuable features under dense occlusion conditions.

**TABLE 5:** The detection results of different density in four architectures.

Evaluation indicator	YOLOv4	YOLOv4-tiny	MobileNet-SSD	Improved YOLOv4
Moderately dense mAP value	89.19%	87.12%	87.28%	89.30%
Highly dense mAP value	83.01%	80.03%	77.16%	84.75%

**FIGURE 9:**
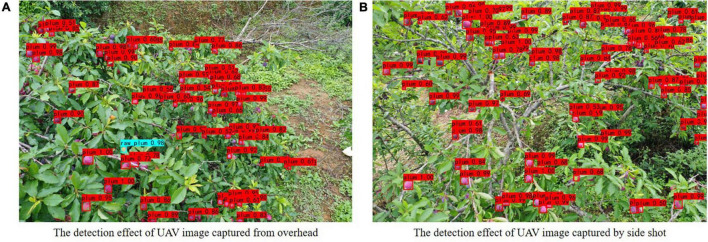
The detection effect of unmanned aerial vehicle (UAV) images.

To further explore the generalization ability of the improved model for image detection in a wide field of view, this study discussed the plum images from unmanned aerial vehicle (UAV) (DJI Yu2, zoom version) at a distance of 2–3 m from the tree canopy and 1–2 m parallel to the plum tree. Then, the improved model was employed to detect and evaluate the collected samples. The detection effect is shown in Figure 9. For the case of dense plums in a large field of view, plums can still be effectively identified by the improved model, indicating that the model has good generalization performance. The conclusion provides the possibility for further research on cooperative picking by UAV and ground fruit-picking robots.

#### A Comparative Experiment of Different Occlusion Situations

There may be some scenes obscured by branches, leaves, and other plums in the natural orchard. These occlusions may affect the detection accuracy of the model. For this reason, we also discussed the detection effect of the improved model on plum images with different occlusion categories.

**FIGURE 10:**
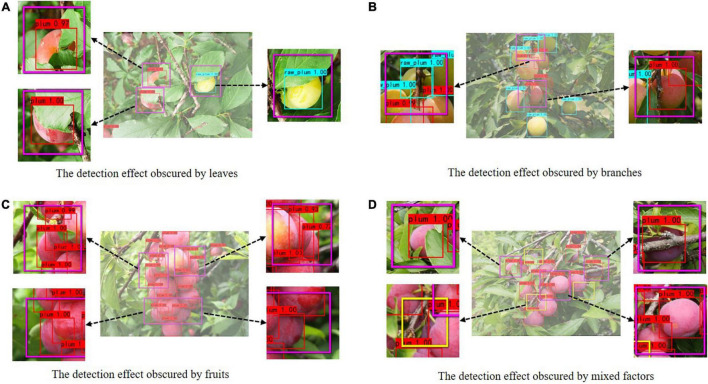
The comparison of plum detection effect under different occlusions.

The detection effect of the improved model for different occlusions is shown in Figure 10. The purple frame represents the partially enlarged image, and the yellow frame indicates the missed plums. As shown in Figures 10A–C, the model can efficiently recognize simple occlusion in the image. As shown in Figure 10D, when there is severe occlusion, plums with large area contour hidden or severely missing texture feature information will be missed. Nevertheless, on the whole, the improved model still has a good recognition effect, which indicates that the introduced Focal Loss function has a certain effect, making the model pay more attention to the occluded and difficult-to-recognize targets during the training process.

## Conclusion

This study focused on dense plums in a real and complex orchard environment and proposed an improved YOLOv4 lightweight model. At first, the plums image data were collected, and the dataset was made using the automatic balancing method based on category and the hybrid offline augmentation method. Then, MobileNetV3 and deep separable convolution were designed to improve the YOLOv4 model, and 152 × 152 feature layers were introduced to deal with the problem of insufficient feature extraction of the dense plums. Withal, the multiscale fusion and the joint loss function of Focal loss and CIOU loss were added to enhance the performance of the model against difficult-to-recognize plums. Finally, the improved model was trained by transfer learning. The main conclusions are as follows:

i. The accuracy of the data automatic balance algorithm proposed in this study for the detection of immature plum reached 86.34%, which is 6.11 percentage points higher than before the imbalance. The mAP increased from 86 to 88.72%, increasing 2.72 percentage points. Overall, the recognition gap of plums with different maturity levels is alleviated, and the robustness of the model is enhanced.
ii. Compared with the other three target detection models, the improved model based on YOLOv4 had the highest mAP result. By comparing with some results from the YOLOv4 model, the model size of the improved model is compressed by 77.85%, the total amount of parameters is only 17.92% of the original model parameters, and the detection speed is accelerated by 112%. The above data show that the improved model has achieved better performance in recognition accuracy and efficiency.
iii. This study discusses the detection performance of the improved model in natural scenes, such as different illuminations, different densities, images collected by UAV, and different occlusion conditions. The experimental results show that the improved model has excellent robustness and generalization performance.

## Data Availability Statement

The original contributions presented in the study are included in the article/supplementary material, further inquiries can be directed to the corresponding author/s.

## Author Contributions

LW designed the experiments and wrote the manuscript. YZ and SL carried out the experiments. LW and YLi collected material data and analyzed experimental results with improved algorithms. SC and YLa supervised and revised the manuscript. All authors contributed to the article and approved the submitted version.

## Conflict of Interest

The authors declare that the research was conducted in the absence of any commercial or financial relationships that could be construed as a potential conflict of interest.

## Publisher’s Note

All claims expressed in this article are solely those of the authors and do not necessarily represent those of their affiliated organizations, or those of the publisher, the editors and the reviewers. Any product that may be evaluated in this article, or claim that may be made by its manufacturer, is not guaranteed or endorsed by the publisher.

## Abbreviations

YOLO: you only look once
CSP: center and scale prediction
DSC: depthwise separable convolution
PWC: pointwise convolution
PANet: path aggregation network
SPP: spatial pyramid pooling
FPN: feature pyramid network
F1: the harmonic mean of the precision and recall
AP: average precision of A category
mAP: average precision of multiple categories
IOU: intersection over union
CIOU loss: complete intersection over union loss
FIOU loss: focal IOU loss
FPS: frame per second
SSD: single shot multibox detector
MobileNet-SSD: MobileNet-single shot multibox detector
UAV: unmanned aerial vehicle.
